# A complex intervention to empower decision-making on mobility among pregnant women during labor: a multi-method study using the MRC framework

**DOI:** 10.1186/s12884-026-09128-8

**Published:** 2026-07-07

**Authors:** Marlene Isabel Lopes, Margarida Vieira, Alexandrina Cardoso

**Affiliations:** 1 Health Sciences Research Unit: Nursing (UICISA: E), School of Nursing, University of Coimbra, Avenida Bissaya Barreto s/n, Coimbra, 3004-011 Portugal; 2 Centre for Interdisciplinary Research in Health: Faculty of Health Sciences and Nursing, Universidade Católica Portuguesa, Porto, Portugal; 3 Nursing School of Porto (ESEP), CINTESIS@RISE: Center for Health Technology and Services Research, Porto, Portugal

**Keywords:** Prenatal education, Decision-making, Labor, Complex intervention, Nurse-midwives, Pregnant women

## Abstract

**Background:**

Freedom of movement and choice of position during labor are associated with improved labor progress, maternal and neonatal outcomes, and more positive birth experiences. However, mobility is often restricted in hospital settings. Antenatal preparation may help women develop the knowledge, confidence, and skills needed to make informed decisions about mobility during labor. This paper reports the development of a complex intervention designed to support such decision-making.

**Methods:**

The intervention was developed in line with the Medical Research Council (MRC) framework for complex interventions. The preparatory phase included a scoping review, in-depth interviews with nurse-midwives and pregnant women, and focus groups with childbirth preparation experts to explore context, needs, barriers, facilitators, and current practices. Findings from these sources were synthesized in a workshop to draft the intervention, followed by a second workshop with researchers and nurse-midwives to refine and validate its structure and content.

**Results:**

The intervention, entitled *I choose to move during my labor*, was developed iteratively with input from nurse-midwives, researchers, experts, and pregnant women attending Childbirth Preparation Programs in Community Care Units. It integrates evidence from the preparatory phase and emphasizes experiential learning, communication, and support for informed decision-making. The final intervention comprises four core components: group sessions, experiential learning activities, decision-making support, and a facilitative communication environment.

**Conclusion:**

This MRC-guided intervention provides a theory-informed and context-sensitive approach to supporting women’s informed decision-making about mobility during labor within antenatal education settings, while offering a foundation for future feasibility testing and implementation.

**Supplementary Information:**

The online version contains supplementary material available at https://doi.org/10.1186/s12884-026-09128-8.

## Introduction

The World Health Organization (WHO) identifies health education as a key component of antenatal care, emphasizing its role in supporting women during pregnancy [1]. Antenatal education programs can help women develop the knowledge, psychological preparation, and practical skills needed for childbirth. These programs may strengthen coping strategies, self-efficacy, and informed choice, with potential benefits for maternal and neonatal outcomes [2]. A positive childbirth experience is a central goal for pregnant women [3]. Active participation in decision-making and a sense of control are important determinants of this experience and strongly influence how women perceive labor and birth [4, 5]. Freedom of movement and the use of upright positions during labor are among the practices that may enhance this sense of control [5]. They are associated with improved labor progression, more effective uterine activity, better maternal-fetal circulation, lower pain perception, and benefits for maternal and neonatal wellbeing [6].

Despite this evidence, restricted mobility and recumbent positions remain common in many hospital settings [7–11]. This gap between evidence and practice limits women’s opportunities to act on their preferences during labor. Although many women wish to participate actively in decision-making [3, 5], research suggests that their decisions are not always fully informed or satisfying, which may undermine self-efficacy and reduce the likelihood of a positive childbirth experience [9, 12].

Decision-making during childbirth is inherently complex. It is shaped by expectations formed during pregnancy, as well as by women’s beliefs, values, and preconceptions [13]. At the same time, intrapartum care may be influenced by risk-averse approaches, institutional routines, and professionals’ values concerning physiological childbirth, all of which can constrain women’s autonomy, even in low-risk situations [14, 15]. Selective or paternalistic information-sharing may further narrow women’s options, while relational and contextual factors, such as rushed interactions or limited professional availability, can weaken confidence in decision-making [13, 14, 16]. Moreover, even when women attend childbirth preparation programs and learn about the benefits of mobility, they may still encounter limited encouragement or insufficient conditions to put this knowledge into practice during labor [9–11]. These issues suggest that antenatal education should go beyond the provision of information alone. It may also need to support women in interpreting information, anticipating barriers, strengthening confidence, and preparing to participate actively in decisions about their bodies and care during labor.

Although the benefits of mobility and upright positions during labor are increasingly recognized, there is limited evidence on how best to support women in making informed decisions about these practices during antenatal preparation. To date, no reviews have specifically focused on interventions designed to strengthen decision-making about mobility and upright positions during labor through antenatal education. This gap highlights the need for theoretically informed and context-sensitive interventions.

Addressing this need, the present paper reports the development of a complex intervention guided by the Medical Research Council (MRC) framework for complex interventions [17, 18]. A multi-method development process, including a scoping review and stakeholder-based qualitative work, informed the intervention. Although the findings from these individual studies are reported elsewhere [19, 20], this paper focuses specifically on how they informed the intervention’s design and final structure. It describes the intervention’s theoretical basis, development process, core components, and refinement, thus providing a clear account of both the development phase and the intervention that emerged from it.

## Methods

A multi-method study was undertaken to develop the intervention, following the development phase of the Medical Research Council (MRC) framework for complex interventions [17, 18]. In line with this framework, the study sought to understand the context, draw on relevant evidence, engage stakeholders, and iteratively refine the intervention design. The development process comprised three stages: preparation, development, and documentation/modeling. Ethical approval for the study was granted by the Health Ethics Committee of the Regional Health Administration Centre on June 22, 2023 (Ref. No. 52/2023). Informed consent was obtained from all participants included in the study.

### Stage 1: preparation

The MRC framework recommends that intervention development begins with an assessment of the relevant evidence base [17]. Accordingly, a scoping review was conducted to identify antenatal education strategies that may support women’s mobility during labor, strengthen self-efficacy, and promote informed decision-making. To complement this evidence and ensure contextual relevance, stakeholder perspectives were also explored through individual and group interviews with nurse-midwives, pregnant women, and experts involved in childbirth preparation programs. These data helped identify needs, barriers, facilitators, and current practices relevant to intervention development.

In summary, the scoping review informed the selection of educational strategies and highlighted the importance of combining information provision with experiential and participatory approaches. Interviews with pregnant women contributed insight into women’s informational needs, beliefs, expectations, and anticipated barriers to using mobility during labor. Interviews and focus groups with nurse-midwives and childbirth preparation experts clarified current antenatal education practices, feasibility constraints, and priorities for implementation within routine care. Together, these sources informed the structure, content, and delivery strategies of the intervention.

### Setting

The intervention was developed for integration into Childbirth Preparation Programs (CPPs) delivered by nurse-midwives in Community Care Units (CCUs), within Portugal’s primary healthcare system. In the Portuguese context, nurse-midwives are specialist nurses in maternal and obstetric health, currently trained at master’s level. CPPs are routinely offered as part of antenatal care and are free of charge. CPPs generally follow a structured antenatal education format, although delivery may vary across units in terms of group size, session format, and local organization. All pregnant women may attend these programs, regardless of their anticipated mode of birth. The intervention was therefore designed to be integrated into the usual CPP structure and to remain relevant across a diversity of women’s expectations, birth plans, and clinical circumstances.

In Portugal, childbirth education is commonly delivered in primary care, whereas labor and birth usually take place in hospital obstetric units. Continuity-of-care models led by nurse-midwives are limited and do not characterize usual public maternity care. Home birth is also a limited option in Portugal and lies outside the usual public maternity care pathway. The intervention was therefore developed with close attention to the hospital contexts in which women are likely to labor and give birth, acknowledging that the use of mobility strategies during labor may depend not only on women’s knowledge and on confidence, but also on the care environment they encounter.

### Stage 2: development

Based on the findings from Stage 1, an initial version of the intervention components and implementation strategies was drafted. Validation workshops were then conducted with the research team and nurse-midwives involved in childbirth preparation. These workshops aimed to review and refine the intervention’s structure, content, delivery format, and feasibility, while considering its contextual relevance and practical applicability within routine antenatal care.

### Stage 3: documentation and modeling

**Fig. 1:**
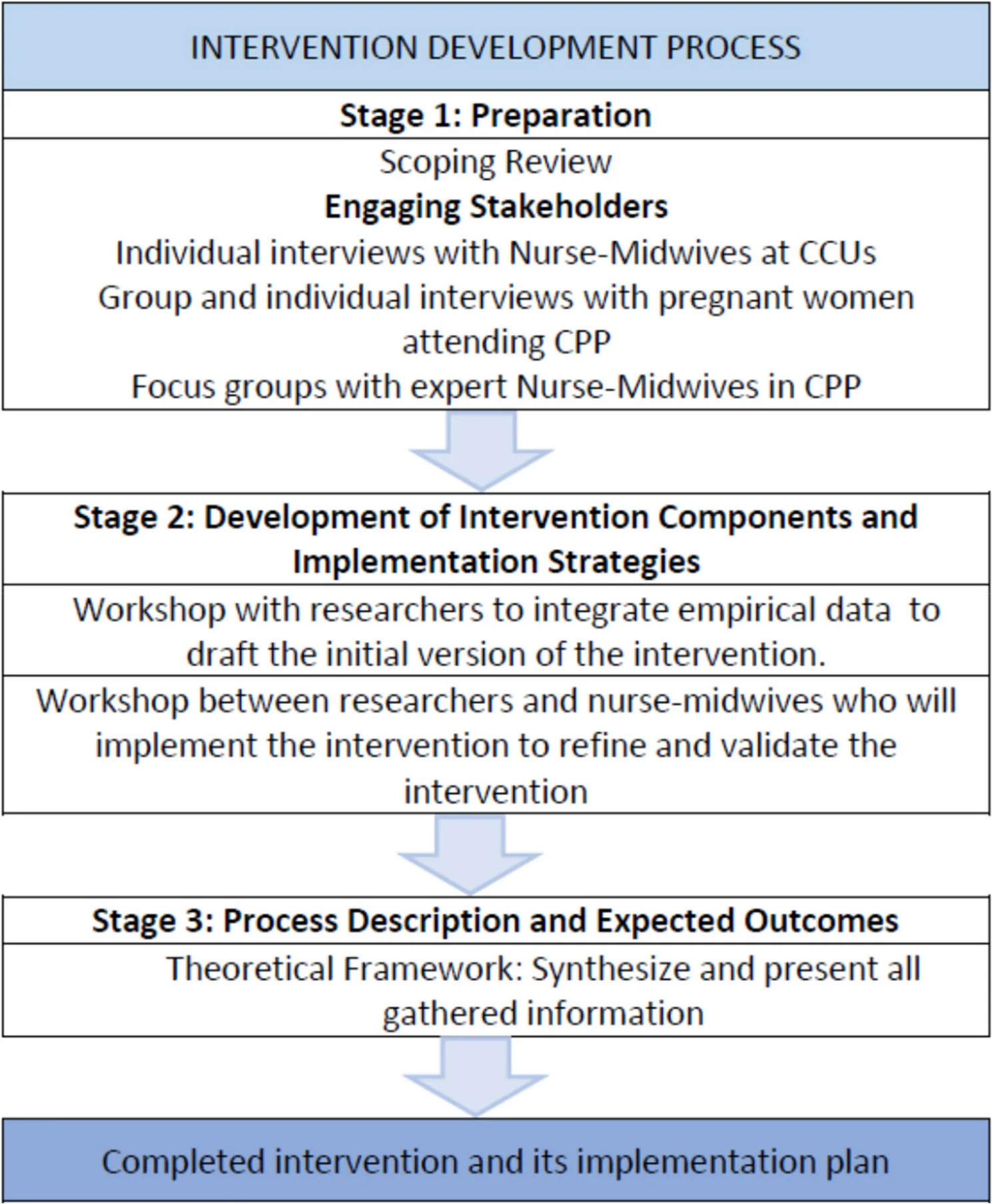
Overview of the development phase

In the final stage, the intervention was documented in detail, including the articulation of its program theory, the development of facilitator guidance, and the preparation of materials to support delivery by nurse-midwives. This stage aimed to ensure that the intervention was systematically described and sufficiently specified to support consistency of delivery and future feasibility testing. Figure 1 provides an overview of the intervention development process.

## Results

The intervention was developed iteratively using evidence from the preparatory phase, stakeholder input, and refinement workshops. This section describes the resulting intervention and the rationale underlying its main components. The reporting of the intervention development process was informed by the GUIDED framework [21].

### Intervention description

#### Objectives and rationale

The intervention was designed to support women in making informed decisions about mobility and upright positions during labor and in preparing to act on those decisions where circumstances allow. It aims to strengthen knowledge, confidence, reflective decision-making, and communication related to mobility during labor, while recognizing that women’s opportunities to use these strategies may also depend on the care environment encountered during childbirth.

Evidence from the preparatory phase indicated that mobility during labor is often constrained by institutional routines, professional practices, and women’s own expectations and beliefs [19, 20]. In response, the intervention was designed not only to provide information about mobility and upright positions, but also to help women reflect on their options, anticipate barriers, and build confidence in expressing and enacting their preferences during labor.

#### Target population and beneficiaries

The intervention was developed for pregnant women attending CPPs, with the possibility of partner involvement. It was designed for use within routine childbirth education and to remain relevant across a diversity of women’s expectations, birth plans, and clinical circumstances.

Nurse-midwives delivering CPPs were also considered key users of the intervention. Their involvement in the development process helped ensure that the intervention was feasible within routine antenatal care and aligned with women’s perceived needs and priorities.

#### Theoretical rationale

The intervention was primarily informed by the theory of emancipated decision-making in women’s health care [12, 22], which emphasizes informed choice, support, and the influence of social norms on women’s healthcare decisions. This framework supported the inclusion of components aimed at strengthening women’s knowledge, reflective decision-making, and ability to express preferences regarding mobility during labor.

Bandura’s self-efficacy theory [23, 24] also informed the intervention design by highlighting the importance of confidence, practice, encouragement, and emotional preparedness. This perspective supported the use of experiential learning, rehearsal of mobility-related strategies, and communication-focused activities intended to help women feel more capable of engaging in decision-making and acting during labor.

#### Intervention components

The final intervention, entitled *I choose to move during my labor*, is a group-based, in-person antenatal program for pregnant women attending CPPs, with the possibility of partner involvement. Four interrelated components were retained following the development and refinement process: (1) in-person group delivery; (2) experiential learning; (3) decision-making training and support; and (4) a safe environment with positive communication.

1. In-person group delivery

Findings from both the scoping review and stakeholder engagement highlighted the value of face-to-face interaction, peer exchange, and opportunities for shared reflection and practice. Women valued direct interaction and the possibility of discussing concerns in a supportive setting, while nurse-midwives and childbirth preparation experts emphasized that social connection among women could strengthen confidence and engagement.

Accordingly, the intervention was designed to be delivered in person and in groups, to facilitate active participation, mutual learning, hands-on practice, and peer support. This format was considered particularly relevant for promoting discussion of labor experiences, normalizing concerns, and supporting women in exploring mobility strategies in preparation for childbirth.

2) Experiential learning

Findings from stakeholders consistently indicated that women needed opportunities not only to receive information, but also to rehearse and reflect on movement-related strategies before labor. Women described the importance of feeling prepared and confident, while nurse-midwives and experts emphasized the value of interactive, practical, and participatory learning methods.

In response, the intervention incorporated observation, simulation, guided practice, and reflective discussion to help women explore mobility and upright positions, adapt them to their own circumstances, and build confidence in their use. Visual materials and take-home guidance were also included to encourage continued practice outside the sessions. This component was intended to support learning through experience, reflection, and rehearsal, rather than through information provision alone.

3) Decision-making training and support

Women often need support to understand their options and to feel able to act on their preferences during labor. Stakeholder findings suggested that women valued strategies that could strengthen their confidence and determination, while nurse-midwives and experts emphasized the importance of preparing women to reflect on available options, recognize sources of pressure, and communicate their preferences in hospital settings where flexibility may be limited.

This component was therefore designed to move beyond information provision by creating structured opportunities for guided reflection and practice in decision-making. Hypothetical scenarios inspired by real-life situations were incorporated to help women consider possible barriers to mobility and upright positions during labor and to reflect on how they might respond in those circumstances. These scenarios address decisions that may influence women’s opportunities for mobility, including admission to hospital, transfer to the labor room, and timing or type of epidural analgesia. To support this process, the intervention incorporates the BRAIN tool, a frequently used decision-support prompt in maternity care [25], to guide consideration of potential benefits, risks, alternatives, personal intuition, and the implications of doing nothing or waiting. Partner involvement was also encouraged in this component to support shared reflection and practical support during labor.

4) Safe environment and positive communication

Findings from both the scoping review and expert consultation highlighted the importance of supportive communication and emotionally safe learning environments in antenatal education. Experts also emphasized that women benefit from opportunities to connect body awareness, movement, relaxation, and confidence-building in preparation for labor.

Accordingly, the intervention incorporated positive communication strategies, conscious breathing, mindfulness-based exercises, and movement-focused relaxation activities to promote calm, bodily awareness, and confidence in using mobility during labor. These activities were designed to support women in engaging with their bodies, practicing movement and positioning in a supportive setting, and developing internal resources that may be helpful during childbirth.

**Fig. 2:**
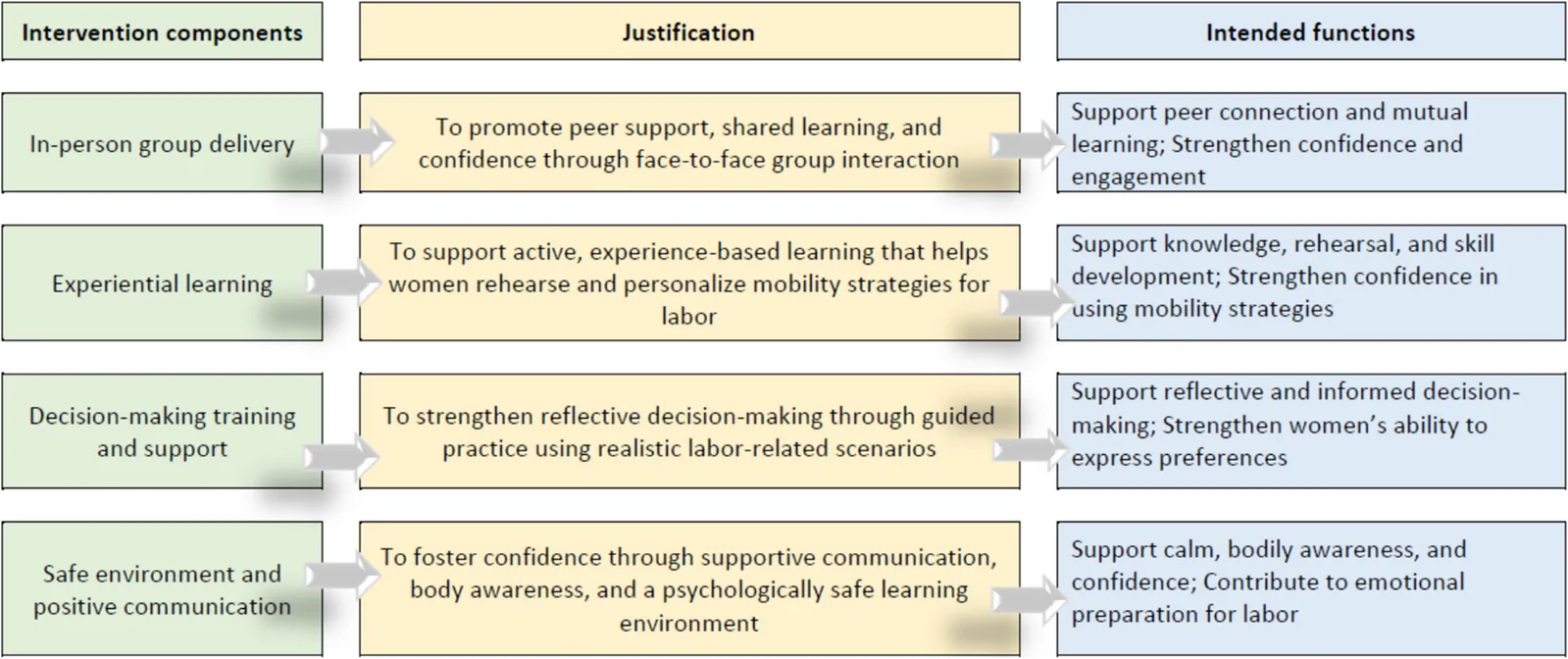
Overview of the intervention components, their justification, and intended functions

Figure 2 illustrates the relationship between the intervention components, their theoretical foundations, and their intended functions.

### Intervention protocol

Building on the evidence base, theoretical framework, and stakeholder consultation, a final intervention protocol was developed. A structured description of the intervention, informed by the TIDieR framework [26], is provided in supplementary material.

An initial version of the intervention (Version 1) was developed based on the scoping review and stakeholder input. During the development phase, uncertainties were identified regarding integration into existing CPPs, session duration and format, and the availability of materials and practical resources. These issues were explored in workshops with participating nurse-midwives. The refinement process indicated that modifying the format from four 1.5-hour sessions to two 2.5-hour sessions was more feasible and better aligned with existing CPP structures. This adaptation was made by condensing and reorganizing content rather than reducing the intervention’s conceptual scope. The core mechanisms of the intervention were retained, namely support for knowledge development, self-efficacy, reflective decision-making, communication, and experiential rehearsal related to mobility during labor. This revised format preserved the intervention’s central aims while making delivery more compatible with routine antenatal care.

The final protocol specifies face-to-face group delivery, with up to eight pregnant women and their birth partners per group. Delivery is undertaken by a nurse-midwife within the existing CPP structure in CCUs. Sessions are designed to take place in a spacious and comfortable room that supports demonstration and hands-on practice. To support consistency of delivery, nurse-midwives are provided with a detailed intervention guide. Participants also receive two informational leaflets: one presenting labor positions and movements that may support comfort and labor progression, and another offering practical suggestions for birth partners on how to provide support during labor. The second leaflet was included because partner attendance at CPPs may not always be possible.

The final intervention comprises two sequential sessions of 2.5 h each. The first session, *Moving during labor - my superpower!*, focuses primarily on experiential learning through training in labor positions and movements to support body awareness and confidence. The second session, *I choose to move during my labor!*, focuses on decision-making training and support through hypothetical scenarios and guided reflection related to mobility and upright positions during labor. Each session includes two segments. The first comprises 100 min of interactive content and practical activities, followed by a 10-minute break. The second comprises 40 min of relaxation-focused activities, combining previously learned labor positions and movements with conscious breathing, mindfulness-based activities, and exercises to strengthen body awareness and emotional preparation. This segment ends with a brief group discussion to consolidate learning and reflection.

Although the intervention was developed primarily as a group-based program, the protocol allows some flexibility in delivery to accommodate participants’ individual needs where appropriate. This was considered important for maintaining a participant-centered approach within routine antenatal care.

## Discussion

This paper describes the development of a complex antenatal intervention designed to support women’s informed decision-making about mobility and upright positions during labor. Guided by the MRC framework [17, 18], the intervention was developed through the integration of theory, evidence, and stakeholder perspectives. This process supported the identification and refinement of components that were conceptually coherent, and feasible for integration into CPP in CCUs.

A key contribution of the development process was the recognition that supporting women’s decision-making about mobility during labor requires more than the provision of information alone. Findings from the preparatory phase indicated that women valued practical preparation, opportunities for reflection, and support in anticipating the challenges they might encounter during labor. This helps explain the inclusion of experiential learning, decision-making training, positive communication, and reflection-based activities as central components of the intervention. These elements are consistent with the theoretical assumptions underpinning the intervention, particularly the importance of strengthening women’s knowledge, confidence, and capacity to express preferences in ways that are personally meaningful and relevant to their circumstances during labor [22].

The findings also reinforced that decision-making during childbirth is shaped not only by individual knowledge and intentions, but also by wider relational, organizational, and cultural influences. Women described concerns about inflexible birth environments, unequal power relations, and the possibility of being judged or receiving less supportive care if they voiced preferences. These concerns echo previous literature on institutional and interpersonal barriers to autonomy in childbirth [13, 16, 27]. For this reason, the intervention was designed not only to inform women about mobility and upright positions, but also to help them reflect on options, recognize sources of pressure, and prepare to communicate preferences in potentially restrictive care environments. At the same time, antenatal preparation alone cannot be expected to overcome all barriers to mobility during labor. Even when women are well informed and motivated, the extent to which mobility is supported may still depend on institutional routines, available resources, professional attitudes, and the flexibility of the intrapartum care setting. This point is important because it situates the intervention within the realities of childbirth care rather than presenting antenatal education as sufficient in itself. In this sense, the intervention may strengthen women’s preparedness and participation, but its practical enactment will remain partly shaped by the care environment they encounter during labor [11, 27–29]. Future feasibility work should therefore attend not only to women’s engagement with the intervention, but also to the contextual conditions that may facilitate or constrain its application in practice.

The intervention was developed for the Portuguese primary care context, in which childbirth education is commonly delivered in CCUs and labor and birth usually occur in hospital obstetric units. In this setting, continuity-of-care models led by nurse-midwives are limited, which makes the transition between childbirth preparation and intrapartum care especially relevant. This context helps explain why the intervention includes components related to communication, reflection, and partner support, in addition to knowledge and practical rehearsal. It also suggests that future implementation may benefit from greater articulation between antenatal educators and hospital-based professionals, particularly if the aim is to support continuity between what women learn antenatally and what they are able to do during labor [27].

The inclusion of a leaflet for birth partners reflects the recognition that support during labor is relational as well as individual. Birth companions can play an important role in reinforcing women’s preferences, providing practical support, and helping sustain confidence during labor [30, 31]. At the same time, their participation in childbirth education may be inconsistent, which justifies the inclusion of take-home materials that extend the intervention beyond session attendance.

Although the intervention was developed for a specific care context, some of its underlying principles may be transferable to other settings. These include the emphasis on experiential learning, reflective preparation for decision-making, supportive communication, and attention to institutional constraints on women’s choices. However, adaptation to other cultural or healthcare contexts would require careful consideration of local maternity care structures, professional roles, childbirth education models, and women’s expectations and beliefs about labor and birth. Language, examples, materials, and delivery formats may all need adjustment to ensure contextual relevance. Although subsequent phases of work have already examined the intervention’s feasibility, acceptability, and preliminary effects [32, 33], the present paper makes a distinct contribution by providing an explicit and transparent account of how the intervention was developed. This remains important because clear reporting of intervention development clarifies how the intervention was shaped, which assumptions underpin it, and which contextual factors may be relevant in future implementation, adaptation, and evaluation.

### Strengths and limitations

A major strength of this study lies in its systematic and theory-informed approach to intervention development. Use of the MRC framework supported attention to evidence, context, stakeholder perspectives, and iterative refinement. The multi-method design also enabled the intervention to be informed by different sources of knowledge, including research evidence, women’s views, and the practical experience of nurse-midwives and childbirth preparation experts. This strengthened the contextual relevance of the intervention and supported the development of components that were both theoretically grounded and practically meaningful. Another strength was the explicit focus on decision-making in relation to mobility during labor, an area that remains underdeveloped in childbirth education research. By combining experiential learning, guided reflection, communication strategies, and attention to self-efficacy, the intervention moves beyond information-based preparation alone and offers a more comprehensive approach to supporting women’s participation in childbirth-related decisions.

Some limitations should also be acknowledged. Although the intervention was informed by multiple stakeholder groups, broader involvement of birth companions and hospital-based professionals might have further strengthened the intervention’s relevance across the transition from community-based antenatal care to hospital-based intrapartum care. In addition, the intervention was developed within a specific organizational and cultural context, which may limit direct transferability to other maternity care systems or childbirth education models. Adaptation to other settings would therefore require further contextual assessment and refinement.

## Conclusion

Using the MRC framework, this study developed a women-centered intervention to support women’s informed decision-making about mobility and upright positions during labor. The development process integrated theory, evidence, and stakeholder perspectives to produce a contextually grounded intervention for use within CPP in CCUs. The final intervention comprises two face-to-face group sessions combining experiential learning, decision-making support, and positive communication strategies. This paper contributes by making the intervention and its development process explicit, thereby supporting transparency, future adaptation, and subsequent feasibility and implementation work.

## Supplementary Information

Supplementary Material 1.

## Data Availability

The data supporting this manuscript were obtained from previously published studies and datasets, all of which have been properly referenced and are publicly accessible. Additional data from studies currently under review or previously cited can be made available upon reasonable request.
