# Supplementary material for: A complex intervention to empower decision-making on mobility among pregnant women during labor: a multi-method study using the MRC framework
Source: BMC Pregnancy Childbirth. 2026 Jul 7;26:1088. doi: 10.1186/s12884-026-09128-8 (PMC13629055; doi:10.1186/s12884-026-09128-8)
Supplement: Supplementary file 1 [file 12884_2026_9128_MOESM1_ESM.docx]

**A complex intervention to empower decision-making on mobility among pregnant women during labor: a multi-method study using the MRC framework**

**Supplementary file**

Table 1. Intervention overview based on the TIDieR Checklist [26]

| Intervention overview based on the TIDieR Checklist | |
| --- | --- |
| ***Brief name*** | *I choose to move during my labor* |
| ***Why*** | Women’s decisions about mobility and upright positions during labor are shaped by multiple interacting factors, including knowledge, beliefs, expectations, confidence, relationships with professionals, and the care environment in which labor takes place. In many settings, institutional routines and limited flexibility may constrain women’s opportunities to act on their preferences. At the same time, women may also feel uncertain about their options, their bodies, and their ability to participate actively in decision-making during labor.  This intervention was developed to address these interrelated influences by supporting women’s informed decision-making about mobility during labor. It aims to strengthen women’s knowledge, confidence, reflective capacity, and ability to express preferences regarding mobility and upright positions, while recognizing that their practical enactment may also depend on the birth setting.  The intervention is informed by two complementary theoretical frameworks. The Theory of Emancipated Decision-Making in Women’s Health Care highlights the importance of informed choice, support, and the influence of social norms and systemic constraints on women’s autonomy. Bandura’s Self-Efficacy Theory contributes an understanding of how confidence, practice, encouragement, and emotional preparedness can shape women’s ability to engage with labor and decision-making. Together, these frameworks support an intervention that combines experiential learning, guided reflection, communication, and confidence-building within antenatal education. |
| ***What*** | *Materials*  The intervention is delivered in a spacious and comfortable room equipped to support demonstration and hands-on practice. Materials include mats, pillows, birthing balls, peanut balls, chairs, an articulated pelvic model, a fetal model to demonstrate labor mechanics, and a marble maze to illustrate fetal descent and positioning.  Nurse-midwives delivering the intervention are provided with an intervention guide to support consistency in delivery. Participants receive two supplementary informational leaflets:  Positioning guide: includes images and explanations of 10 labor positions and movements that may support comfort and labor progression.  Support partner tips: provides practical suggestions to help birth partners offer effective support during labor.  *Procedures*  The intervention is offered within Childbirth Preparation Programs (CPPs) in Community Care Units (CCUs) and is intended for pregnant women in the third trimester, with the possibility of partner involvement. The total duration is approximately 5 hours, divided into two structured sessions of 2.5 hours each.  *Session I: Moving During Labor – My Superpower!*   - Part 1: development of knowledge and body awareness through hands-on exploration of labor positions and movements. - Part 2: relaxation-focused activities combining previously introduced positions and movements with conscious breathing, mindfulness-based activities, and exercises intended to strengthen body awareness and emotional preparation.   *Session II: I Choose to Move During Labor!*   - Part 1: decision-making training and support through hypothetical scenarios and guided reflection on mobility and upright positions during labor. - Part 2: relaxation-focused activities that integrate movement, positioning, and comfort strategies, followed by a brief group discussion to consolidate learning and reflection.   *Key features of the intervention*  Positive communication: nurse-midwives use supportive and encouraging communication strategies to foster participation, reflection, and confidence within the group.  Experiential learning and reflection: participants are actively engaged in practicing positions and movements, observing demonstrations, and reflecting individually and collectively on their relevance to labor.  Integration of movement and relaxation: labor positions and movements are practiced together with conscious breathing, mindfulness-based activities, and body-awareness exercises to support preparation for labor. |
| ***Who provided*** | The intervention is delivered by a nurse-midwife within the existing Childbirth Preparation Program in Community Care Units. In the study context, participating nurse-midwives had more than 5 years of experience in conducting childbirth preparation programs. |
| ***How*** | The intervention is delivered face-to-face in a group format, with up to 8 pregnant women and their birth partners per group. This format is intended to support interaction, peer exchange, and active participation. Two informational leaflets are provided to reinforce learning and offer practical guidance for labor positions and birth partner support. Although the intervention was developed primarily as a group-based program, the protocol allows limited flexibility in delivery where needed to accommodate participants’ individual circumstances. |
| ***Where*** | The intervention is delivered in a room that can comfortably accommodate up to 8 pregnant women and their birth partners and support demonstration and hands-on practice. The space is organized to allow movement-based activities and includes materials such as mats and birthing balls. A private, comfortable, and interruption-free environment is considered important to support participation, communication, and experiential learning. |
| ***When and How Much*** | The intervention is integrated into the Childbirth Preparation Program during the third trimester of pregnancy. The total duration is approximately 5 hours, delivered across two sequential sessions of 2.5 hours each. The timing of delivery within the broader Childbirth Preparation Program may be adapted to fit the organization of each program, while retaining the intervention’s two-session structure.  Each session is structured in two segments:  First segment: 100 minutes of interactive content and hands-on practice  Break: 10 minutes  Second segment: 40 minutes of focused activities, including relaxation-based practice and concluding group discussion |
| ***Tailoring*** | The intervention was developed primarily as a group-based program, but the protocol allows some flexibility in delivery to accommodate participants’ individual needs where appropriate. This may include adjusting the emphasis placed on particular mobility strategies, relaxation-based activities, or reflective discussions according to participants’ circumstances, preferences, or learning needs. This flexibility was considered important for maintaining a participant-centered approach within routine antenatal care while preserving the core structure and aims of the intervention. |
| ***Modifications*** | The initial version of the intervention (Version 1) was developed using insights from the scoping review and stakeholder input. During this phase, uncertainties were identified regarding integration into existing Childbirth Preparation Programs, overall duration, delivery format, and the availability of materials and practical resources. These issues were explored in workshops with participating nurse-midwives. Through this refinement process, the intervention format was modified from four 1.5-hour sessions to two 2.5-hour sessions in order to improve feasibility and alignment with existing Childbirth Preparation Program structures. This change involved condensing and reorganizing the content rather than reducing the intervention’s conceptual scope. The core mechanisms of the intervention were retained, including support for knowledge development, self-efficacy, reflective decision-making, communication, and experiential rehearsal related to mobility during labor. This revised format preserved the intervention’s central aims while making delivery more compatible with routine antenatal care. |
| ***How Well*** | To support consistency of delivery, nurse-midwives are provided with an intervention guide and opportunities for clarification and feedback where needed. In subsequent feasibility work, attention may be given to fidelity of delivery, implementation challenges, and contextual adaptations. |
